# Rapid diagnosis of pulmonary tuberculosis on induced sputum in children in a high burden setting for HIV and malnutrition

**DOI:** 10.1371/journal.pgph.0007271

**Published:** 2026-10-01

**Authors:** Juaneta Luiz, Lesley J. Workman, Cynthia B. Baard, Yekiwe Hlombe, Margaretha Prins, Nomlindo Makubalo, Devan Jaganath, Adithya Cattamanchi, Mark P. Nicol, Heather J. Zar

**Affiliations:** 1 Department of Paediatrics and Child Health, University of Cape Town, and South African Medical Research Council Unit on Child & Adolescent Health, Red Cross War Memorial Children’s Hospital, Cape Town, South Africa; 2 Department of Paediatrics, Dora Nginza Hospital, Gqeberha, South Africa; 3 Division of Pediatric Infectious Diseases, University of California San Francisco, San Francisco, United States of America; 4 Center for Tuberculosis, Institute for Global Health Sciences, University of California San Francisco, San Francisco, United States of America; 5 Division of Pulmonary Diseases and Critical Care Medicine, University of California Irvine, Irvine, United States of America; 6 Division of Medical Microbiology and Institute for Infectious Diseases and Molecular Medicine, University of Cape Town and National Health Laboratory Services, Cape Town, South Africa; 7 Marshall Centre, School of Biomedical Sciences, University of Western Australia, Perth, Australia; PLOS: Public Library of Science, UNITED STATES OF AMERICA

## Abstract

Microbiological diagnosis of childhood pulmonary tuberculosis can be challenging. We investigated the accuracy of Xpert MTB/RIF Ultra on the induced sputum, focused on high-risk groups (infants, malnourished children, and those living with HIV), where data is limited. Children (under 15 years) investigated for pulmonary tuberculosis at Dora Nginza Hospital, South Africa, were prospectively enrolled between January 2018 and January 2024, and followed up for 12 months. Induced sputa were tested with liquid culture and Xpert MTB/RIF Ultra (Ultra). Children were categorized with confirmed, unconfirmed, or unlikely tuberculosis, according to NIH consensus definitions. Ultra diagnostic accuracy was calculated against culture, with analysis by high-risk subgroups. Of 636 children (median age, 27.4 (IQR 11.8–61.6) months), 173 (27.2%) were classified with confirmed TB, 384 (60.4%) with unconfirmed TB, and 79 (12.4%) with unlikely TB. There were 223 (37.8%) children underweight-for-age, 168 (26.5%) living with HIV, and 163 (25.6%) infants. Of 557 children with TB, 145 (26.0%) were confirmed on induced sputa; with 137/557 (24.6%) confirmed on Ultra (46.0%; 63/137, of which were “trace positive”), and 88/557 (15.8%), on culture. Of those confirmed, 57/145 (39.3%) were Ultra positive, culture negative – contributing 10.2% to the overall yield. Ultra performed better in children over 5 years and with weight-for-height Z-scores < -3. Overall, Ultra sensitivity was 90.9% (95%CI 82.9-96.0) and specificity, 89.6% (95%CI 86.7-92.0), with high accuracy across high-risk groups. Mortality amongst children treated for PTB was 4.9% (27/557) with aRR highest for stunted children (7.3; 95% CI, 2.2-24.2), followed by children with HIV (3.5; 95% CI, 1.6-7.5), and infants (3.3; 95% CI, 1.6-6.7). Ultra on induced sputum was feasible, with high yield and diagnostic accuracy for high-risk children.

## Introduction

An estimated 1.1 million children are diagnosed with tuberculosis (TB) disease annually, although underdiagnosis and under-reporting are concerns [1]. Mathematical modelling estimates that almost 60% of cases are unreported in high burden countries, with 25% of global incident TB cases attributed to undernutrition, HIV infection, or BCG non-vaccination [1,2]. Approximately 80% of childhood TB deaths occur in children under 5 years, and 96% of overall childhood mortality occurs in those not on treatment, underscoring serious diagnostic gaps [1,3]. Non-specific clinical and radiological findings add to the diagnostic challenge [1,3].

Young children, children living with HIV (CLHIV), and those with malnutrition, are at high risk for severe disease and mortality [1]. Attaining a microbiological diagnosis may be difficult due to the paucibacillary nature of childhood TB disease and the inability to spontaneously expectorate [4]. However, improved sample collection methods and rapid diagnostics have increased diagnostic yield. Several studies have shown that sputum induction is safe and feasible even in infants, providing a sample that currently has the highest diagnostic accuracy, particularly when testing with *Xpert MTB/RIF Ultra* (Ultra) [4–8]. Despite this, the induced sputum (IS) procedure has not been widely implemented in high-burden settings, with perceived concerns around feasibility and accuracy particularly in high-risk groups, and with most data arising from well-resourced tertiary settings in high burden countries [4–9].

South Africa has one of the highest overall TB incidences worldwide (389/100 000 in 2024), and an estimated childhood TB case notification rate of 165 per 100’000 in 2016 - almost half of whom were under the age of 5 years [10,11]. Close to 86% of all CLHIV live in sub-Saharan Africa, with the estimated South African HIV incidence rate over six times that of the global average (1.16 versus 0.18 per 1000 children aged 0–14 years) [12,13]. Malnutrition is common with 27% of children under five years stunted nationally, and severe acute malnutrition (SAM) reported as an underlying cause in 14% of all-cause childhood mortality [14,15].

There are limited data on optimal strategies for the diagnosis of pulmonary TB (PTB), and diagnostic accuracy of molecular testing in children at high risk of severe disease and mortality, particularly, infants, CLHIV, or malnourished children. The primary aim of this study was to investigate the diagnostic accuracy of Ultra compared to culture using induced sputum sampling in high-risk children.

## Methods

### Ethics statement

The study was approved by the University of Cape Town Human Research Ethics Committee (HREC Reference number: 045/2008) for the duration of the study. Additional committee approvals were granted through Dora Nginza Hospital and through the National Health Research Database for the Eastern Cape Province in South Africa.

### Setting and eligibility

A prospective study of children aged 0–15 years with signs and symptoms suggestive of PTB attending Dora Nginza Hospital, Gqeberha, was conducted from 09 January 2018–19 January 2024. This hospital offers both secondary and tertiary pediatric care (including high-volume out-patient services) to a population of over two million in the under-resourced Eastern Cape province of South Africa [16]. Prior to study initiation, the study team conducted practical training on IS and childhood TB diagnostics, as a robust microbiological childhood TB diagnostics program was lacking at this facility and at referral clinics.

Children were enrolled if they presented with a cough of at least two weeks, or a cough of any duration with one or more of: failure to thrive, poor weight gain, or loss of weight; a positive tuberculin skin test (TST); a chest radiograph suggestive of PTB; or close exposure to an individual with TB disease. A TST induration of ≥5mm was considered positive for CLHIV or those with severe malnutrition as per WHO guidelines, and ≥10mm was considered positive for all other children [17]. Children were excluded if they received TB treatment or prophylaxis for more than 72 hours, if they were treated for TB within the previous 12 months (for children enrolled from August 2021), or where follow up was not possible. Both ambulatory and hospitalized children were screened for enrolment by study staff, along with children and caregivers interested in joining the study who were referred by healthcare workers from Dora Nginza Hospital and primary care facilities in the Nelson Mandela Bay Metro. Written informed consent was obtained from a legal guardian, and written assent was obtained from children older than seven years.

### Procedures and definitions

A history, anthropometry, and clinical examination were performed, and a TST, placed and read at 48–72 hours. HIV exposure was noted, with HIV testing done where the status was unknown. Children were followed at one and three months, at treatment completion (for those on TB treatment), and at 12 months, to monitor clinical response and confirm TB diagnostic category based on NIH consensus definitions for confirmed, unconfirmed, and unlikely TB [18].

We defined nutritional status based on WHO classifications using weight-for-age Z-scores (WAZ) for children 0–10 years, height-for-age Z-scores (HAZ) for children 0–15 years, weight-for-height Z-scores (WHZ) for children 0–5 years, and body mass index-for-age (BMI-for-age) Z-scores for children 5–15 years [19].

The IS was performed as previously described [8]. Following at least three hours of fasting, IS was obtained by study or trained hospital staff. Induction involved pre-treatment with an inhaled bronchodilator, followed by sterile hypertonic saline (5%) nebulized with oxygen at a flow rate of 5-8L/min for 2–5 minutes. Sputum was then obtained via expectoration for children able to adequately expectorate. For those unable, the nasopharynx was suctioned using a sterile mucus extractor kit. Pulse oximetry was monitored pre- and post-procedure.

Samples were transported on ice to the local accredited TB laboratory and processed within 24–72 hours using standardized protocols as per manufacturer instruction for *Xpert MTB/RIF Ultra* and automated liquid culture (*Becton Dickinson Bactec MGIT 960*). Laboratory staff performing and reviewing culture results were blinded to smear and Ultra results, and vice versa for those performing smear and Ultra. Additional methodological detail is provided in the online data supplement.

### Statistical analysis

Data were analyzed using STATA 19.5 (STATA Corporation, College Station, TX, USA). A convenience sample size of all consecutively enrolled children with at least one valid Ultra and culture result on the same IS were included. Kruskal-Wallis, Wilcoxon rank-sum, Chi-squared, or Fisher’s exact tests were used for crude comparisons as appropriate. Sensitivity, specificity, and predictive values were calculated using culture positive on IS as the reference standard. Generalized linear models specifying a Poisson distribution and logarithmic link function with robust standard errors were used to determine differences between high-risk groups: for factors including nutritional status, tuberculin skin test positivity, and hospitalisation, and for baseline Ultra positivity and mortality at any time over the follow-up period. For interpretation, the exponentiated regression coefficients are reported as Risk Ratios (RR) and adjusted Risk Ratios (aRR) with their corresponding 95% confidence intervals (95% CI).

Statistical tests were two-sided; a p-value ≤0.05 was considered statistically significant.

## Results

### Study population

There were 700 children enrolled; IS was successfully performed in all but 17 children with severe respiratory distress. A further 47 were excluded predominantly due to a lack of Ultra and culture results on the same IS sample, or loss to follow-up, providing 636 children for the analysis (Fig 1). Overall, 173 (27.2%) children were classified with confirmed TB on any sample type, 384 (60.4%), with unconfirmed TB, and 79 (12.4%), with unlikely TB. Among those confirmed, 145/173 (83.8%) were confirmed on IS, 10/173 (5.8%), on another respiratory sample (four gastric aspirates, three endotracheal aspirates, two nasopharyngeal aspirates, and one spontaneously expectorated sputum), and 18/173 (10.4%), on an extra-pulmonary sample only (including lymph node and abscess aspirates, cerebrospinal fluid, and pericardial fluid).

**Fig 1:**
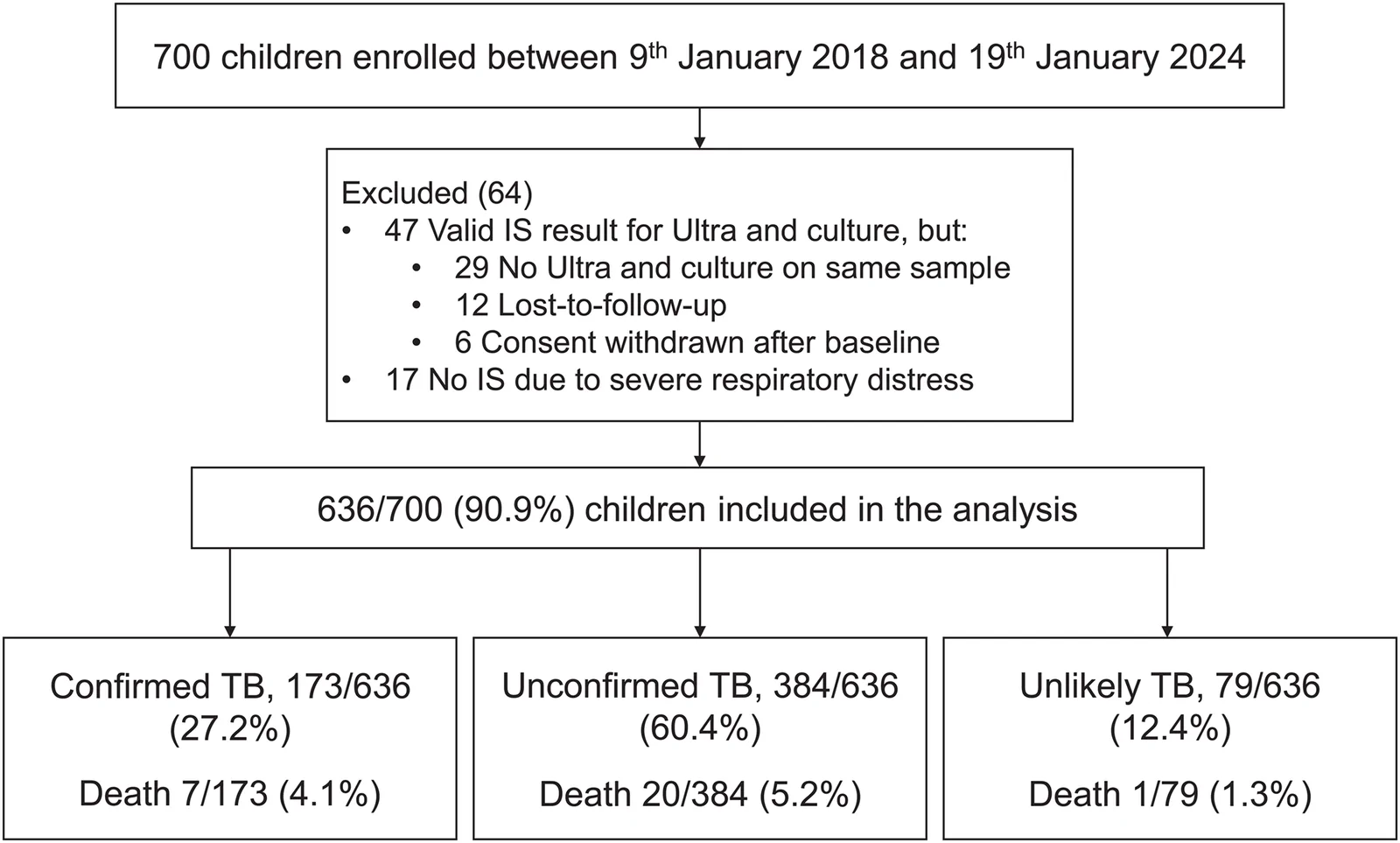
Flow diagram of participants. “Confirmed TB” in this diagram refers to children with a positive result for Ultra and/or culture on any sample type (respiratory and/or extrapulmonary TB). IS = Induced Sputum.

Of 636 children, 385 (60.5%) were hospitalized, and 163 (25.6%) were infants. Malnutrition was highly prevalent with 223/590 (37.8%) children underweight-for-age (WAZ < -2), 268/632 (42.4%), stunted (HAZ < -2), and severe malnutrition noted in 68/467 (14.6%) children aged 0–5 years (WHZ < -3) and 20/165 (12.1%) children aged 5–15 years (BMI-for-age < -3; Table 1). HIV exposure occurred in 50.9% (323/634) of children; with 24.5% (155/634) HIV exposed but uninfected (HEU), and 26.5% (168/634) living with HIV. Of CLHIV, 140 (83.3%) were previously diagnosed with HIV, of whom 67.1% (94/140) were on antiretroviral therapy at the time of enrolment.

**Table 1:** Baseline characteristics, and HIV and nutritional status, of children by TB category.

Variable	All	Confirmed TB*	Unconfirmed TB	Unlikely TB	p-value
Number of Children	**636**	**173 (27.2)**	**384 (60.4)**	**79 (12.4)**	
Hospitalized	385 (60.5)	100 (57.8)	238 (62.0)	47 (59.5)	0.634
Median (IQR) age in months	27.4 (11.8-61.6)	30.0 (11.8-85.3)	27.3 (12.7-58.5)	21.2 (9.4-46.0)	0.056
Age categories <1 year	163 (25.6)	45 (26.0)	92 (24.0)	26 (32.9)	
≥1 to <5 yr	308 (48.4)	68 (39.3)	202 (52.6)	38 (48.1)	0.008
≥5 years	165 (25.9)	60 (34.7)	90 (23.4)	15 (19.0)	
Sex (% male)	336 (52.8)	96 (55.5)	199 (51.8)	41 (51.9)	0.713
TST positive (n = 558)	266 (47.7)	77 (52.4)	170 (50.0)	19 (26.8)	0.001
TB contact (n = 634)	351 (55.4)	92 (53.2)	221 (57.9)	38 (48.1)	0.226
HIV^†^ (n = 634)					
HIV unexposed, uninfected	311 (49.1)	96 (55.5)	167 (43.7)	48 (60.8)	
HIV exposed, uninfected	155 (24.5)	38 (22.0)	92 (24.1)	25 (31.7)	<0.001
CLHIV	168 (26.5)	39 (22.5)	123 (32.2)	6 (7.6)	
CD4 count median (IQR)^‡^, cells per microliter	577 (225-1048)	437 (113-661)	689 (318-1121)	218 (132-1337)	0.017
CD4% median^‡^ (IQR)	18.4 (10.4-29.4)	16.9 (6.7-24.9)	19.4 (10.7-29.6)	12.0 (9.4-19.3)	0.472
Nutrition^§^					
Underweight-for-age (WAZ < -2)^‖^ (n = 590)	223 (37.8)	63 (42.3)	142 (38.9)	18 (23.7)	0.019
Severely underweight-for-age (WAZ < -3)^‖^ (n = 590)	122 (20.7)	37 (24.8)	77 (21.1)	8 (10.5)	0.041
Stunted (HAZ < -2)^§^** (n = 632)	268 (42.4)	81 (46.8)	163 (42.7)	24 (31.2)	0.068
Severe stunting (HAZ < -3)^§^** (n = 632)	128 (20.3)	38 (22.0)	81 (21.2)	9 (11.7)	0.134
Severely malnourished; WHZ < -3^§††^ (n = 467)	68 (14.6)	21 (18.6)	42 (14.4)	5 (8.1)	0.167
Severely malnourished; BMI-for-age < -3^§^**^‡‡^ (n = 165)	20 (12.1)	9 (15.0)	10 (11.1)	1 (6.7)	0.615

Data reported as number (proportion) of the total number for categorical data and median (IQR) for continuous data. IQR = interquartile range. TST = tuberculin skin test. WAZ = weight-for-age Z-score, HAZ = height-for-age Z-score, WHZ = weight-for-height Z-score. CLHIV = children living with HIV.

* “Confirmed TB” in this table refers to children with a positive result for Ultra and/or culture on any sample type (respiratory and/or extrapulmonary TB).

† HIV status unknown for two children.

‡ Four CLHIV with CD4 results unknown.

§ Four heights unknown (excluded from HAZ, WHZ and BMI-for-age calculations).

**‖** WAZ calculations based on WHO Z-scores for weight-for-age for children 0–10 years.

** HAZ calculations based on WHO Z-scores for height-for-age for children 0–15 years.

†† WHZ calculations based on WHO Z-scores for weight-for-length or weight-for-height for children 0–5 years.

‡‡ BMI-for-age calculations based on WHO Z-scores for BMI-for-age for children 5–15 years.

Amongst children diagnosed with PTB (with confirmed or unconfirmed TB), infants were particularly at increased risk of being underweight-for-age, and at twice the risk of being hospitalized than older children (Table 2). Similarly, compared to HIV negative children, CLHIV were at almost twice the risk of being underweight-for-age or stunted, and over twice the risk of having a negative TST (Table 2). Children that were underweight-for-age were at almost twice the risk of being hospitalized (Table 2). Amongst children diagnosed with PTB, hospitalization rates were similar between CLHIV (95/162; 56.2%) and HIV negative children (245/393; 62.3%, p = 0.176).

**Table 2:** Factors associated with high-risk groups amongst children investigated for PTB.

	Infants <1 year (n = 163), vs > 1 year for RR	CLHIV* (n = 168), vs HIV negative for RR	WAZ^†^ < -2 (n = 223), vs WAZ ≥ -2 for RR
**WAZ^†^ < -2 (%)**	82/163 (50.3)	80/144 (55.6)	..
RR (95% CI)	**1.7 (1.3-2.3)**	**1.9 (1.4-2.5)**	**..**
p-value	<0.001	<0.001	..
**HAZ^‡§^ < -2 (%)**	82/160 (51.3)	99/168 (58.9)	..
RR (95% CI)	**1.4 (1.1-1.9)**	**1.8 (1.4-2.4)**	**..**
p-value	<0.016	<0.001	..
**WHZ^‡‖^ < -3**	34/160 (21.3)	15/99 (15.2)	..
RR (95% CI)	**1.5 (1.1-2.1)**	**1.0 (0.6-1.7)**	**..**
p-value	0.006	0.812	..
BMI-for-age^‡^** < -3	..	12/69 (17.9)	..
RR (95% CI)	**..**	**1.5 (1.0-2.2)**	**..**
p-value	..	0.045	..
**TST negative (%)**	88/145 (60.7)	91/133 (68.4)	115/192 (59.9)
RR (95% CI)	**1.4 (1.0-1.9)**	**2.1 (1.6-3.0)**	**1.4 (1.1-1.8)**
p-value	0.051	<0.001	0.006
**Hospitalized (%)**	123/163 (75.5)	95/168 (56.6)	163/233 (73.1)
RR (95% CI)	**2.0 (1.4-2.9)**	**0.8 (0.6-1.1)**	**1.8 (1.4-2.3)**
p-value	<0.001	0.175	<0.001

Data is reported as number (proportion) of the total number of children for the selected high-risk group column that have the outcomes noted in the corresponding row (where data is available, and relevant - based on age). Risk Ratios (RR) refer to the risk ratio for those in each high-risk group column (infants, children living with HIV, and those under-weight-for age) to have the outcome based on the corresponding row title, compared to those: older than one year old, not living with HIV, and not under-weight-for-age respectively, with associated p-values. WAZ = WHO weight-for-age Z-score, HAZ = WHO Height-for-age Z-score < -2, WHZ = WHO weight-for-length or weight-for-height Z-score, TST = Tuberculin Skin Test; CLHIV = Children Living with HIV; data reported as number (proportion), univariate generalized linear models were used to generate the risk ratios (RR) and 95% confidence intervals (CI).

* HIV status unknown for two children.

† WAZ calculations based on WHO Z-scores for weight-for-age for children 0–10 years.

‡ Four heights unknown (excluded from HAZ, WHZ, and BMI-for-age calculations).

§ HAZ calculations based on WHO Z-scores for height-for-age for children 0–15 years.

**‖** WHZ calculations based on WHO Z-scores for weight-for-length and weight-for-height for children 0–5 years.

**BMI-for-age calculations based on WHO Z-scores for BMI-for-age for children 5–15 years.

A previous episode of TB disease was reported in 56/630 (8.9%) children, with 12/630 (1.9%) children having episodes within the past year.

### Induced sputum diagnostic accuracy, safety and yield

Of 1051 IS samples collected from 636 children, all had a valid microbiological result for at least one sample. The youngest participant was 30 days old. Hospital staff collected 452/636 (71.1%) of the first IS samples. No serious adverse events occurred during sputum induction; minor adverse events occurred in 23/636 (3.6%) children, including 22 with mild epistaxis and one with vomiting, all of which resolved spontaneously.

Of the 557 children diagnosed with PTB, 145 (26.0%) had microbiological confirmation on IS. Of these, 137/145 (94.5%) were Ultra positive and 88/145 (60.7%), culture positive. This provided a per patient yield of 24.6% (137/557) for Ultra and 15.8% (88/557) for culture.

Overall, Ultra sensitivity per child (n = 636) was 90.9% (95%CI, 82.9-96.0), and specificity, 89.6% (95%CI, 86.7-92.0) against IS culture, considering Ultra and culture results for all IS samples taken for each child (S1 Table). Ultra diagnostic accuracy per child for the first IS (IS1) against any IS culture was high across risk groups (Fig 2, S1 Table).

**Fig 2:**
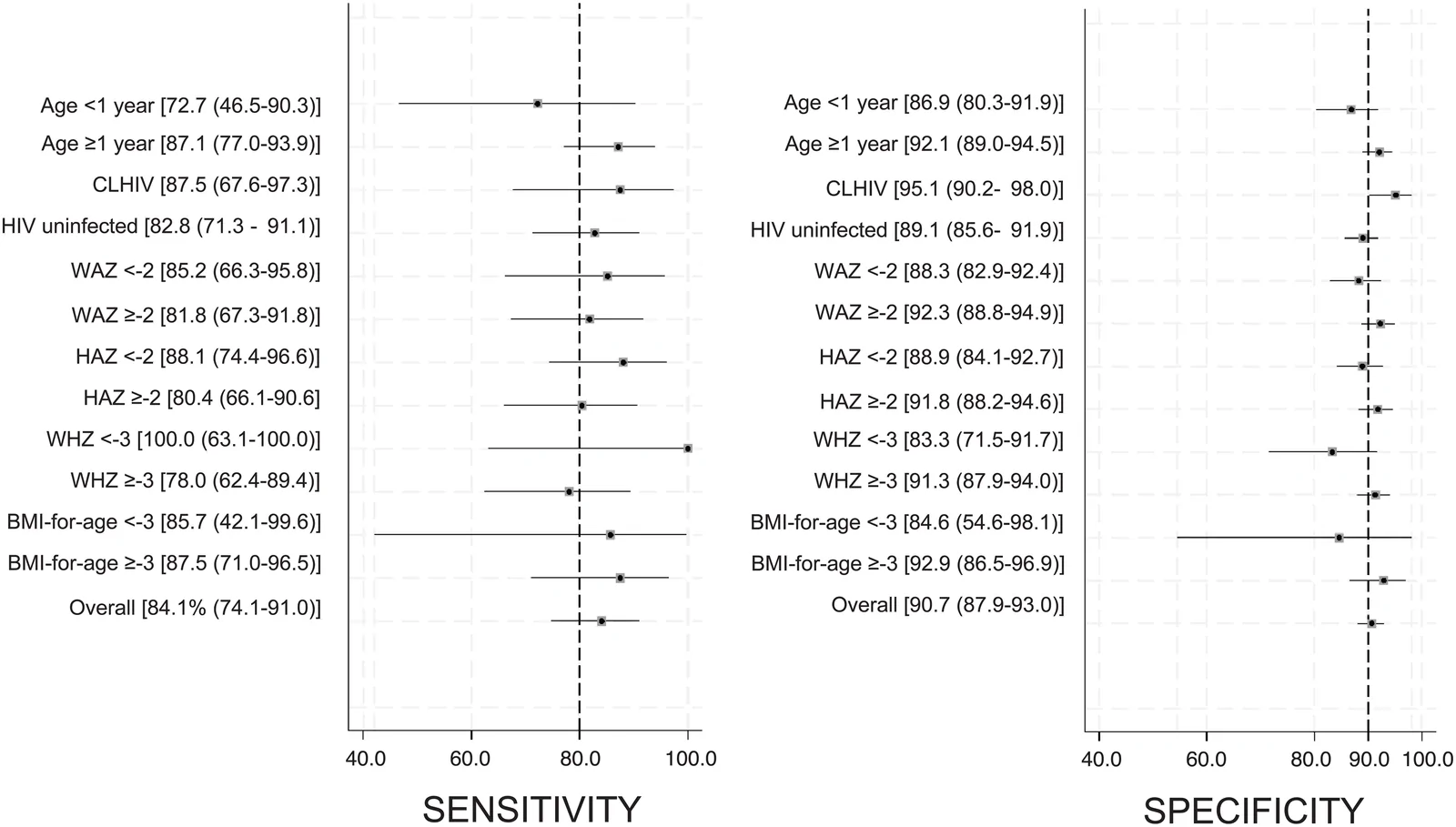
Forest plot of sensitivity and specificity (%), with 95% Confidence intervals, of first induced sputum Ultra compared to culture on any induced sputum, across risk-groups. WHZ = WHO weight-for-length or weight-for-height Z-score, HAZ = WHO height-for-age Z-Score, WAZ = WHO weight-for-age Z-score, CLHIV = Children Living with HIV.

Of 137 children with a positive Ultra on IS, a “trace positive” result occurred for 63 (46.0%) children, of which 55 (87.3%) were under ten years old, and seven (11.1%) had previous TB.

Of 88 children with a positive culture, the corresponding Ultra was positive in 90.9% (80/88). An additional 57 culture negative children had a positive IS Ultra; of these, 71.9% (41/57) were “trace positive.” These 57 children contributed 10.2% to the overall yield; increasing from 15.8% (88/557) diagnosed by culture alone, to 26.0% (145/557) diagnosed overall. Children who were Ultra positive, but culture negative were significantly younger than those both Ultra and culture positive (S3 Table). Five out of 57 children (8.8%) had a previous episode of TB (one within the prior year). All children diagnosed with PTB had clinical and/or radiological evidence for disease and improved on TB treatment.

Children diagnosed with TB disease older than five years had a higher IS1 Ultra yield (29.3%; 44/150) than younger participants (19.9%, 81/407), p = 0.018. HIV negative children had a higher IS1 Ultra yield (27.4%), compared to those HIV exposed but uninfected (19.2%), and CLHIV (17.3%); p = 0.032 (Fig 3). For children with a WHZ < -3 (aged 0–5 years), aRR for IS1 Ultra positivity was 1.7 (1.1-2.6), compared to those WHZ ≥ -3, adjusted for HIV status and age (p = 0.027; S4 Table). Conversely, amongst CLHIV (aged 0–5 years), the aRR for IS1 Ultra positivity was 0.3 (0.1-0.7) compared to HIV negative children, adjusted for WHZ and age (p = 0.003; S4 Table).

**Fig 3:**
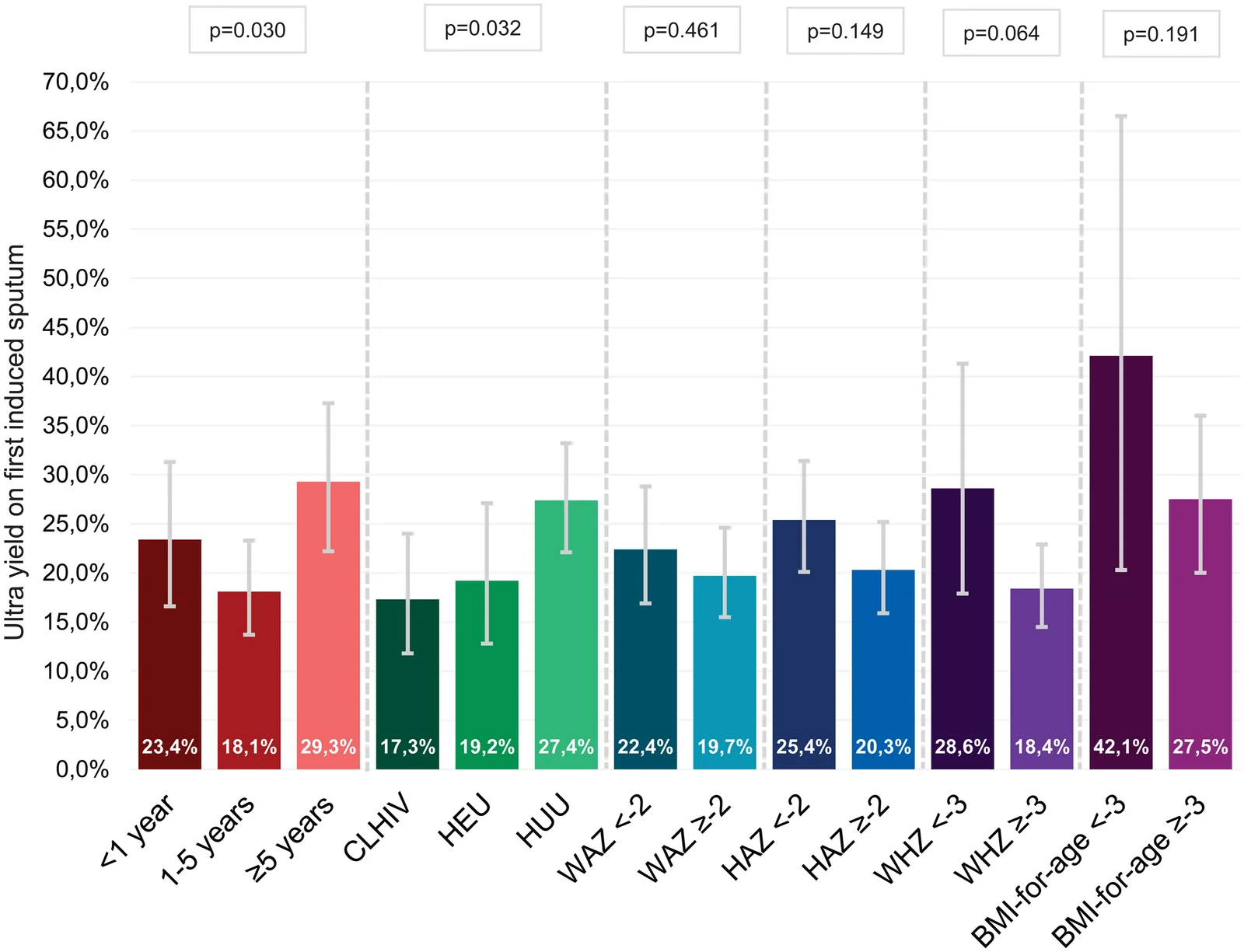
Ultra yield (proportion testing positive, with 95% confidence intervals) from first induced sputum specimen, by risk groups, amongst children diagnosed with PTB. HEU = HIV exposed and uninfected, HUU = HIV unexposed and uninfected, CLHIV = Children Living with HIV, WAZ = WHO weight-for-age Z-score, HAZ = WHO height-for-age Z-Score, WHZ = WHO weight-for-length or weight-for-height Z-score. Chi-squared test was used to explore differences in proportion positive for each risk group separately; WAZ < -2 vs WAZ ≥ -2, HAZ < -2 vs HAZ ≥ -2, WHZ < -3 vs WHZ ≥ -3, BMI-for-age < -3 vs BMI-for-age ≥ -3.

### Drug resistance

There were 27/557 (4.9%) children diagnosed with drug-resistant TB; 15 (55.6%) based on exposure to a close TB contact, and 12 (44.4%), on laboratory results - making up 8.3% (12/145) of those with confirmed TB. Similarly, two (2/145; 1.4%) had confirmed isoniazid mono-resistant TB, and 10/145 (6.9%), rifampicin-resistant TB (RR-TB) comprising four each with rifampicin-mono-resistant (RMR) and multi-drug resistant (MDR-TB), and two with rifampicin-resistant TB where other drug resistance is unknown. Of those with RR-TB, one was diagnosed on gastric aspirate, and nine, on IS - of which 7/9 (77.8%) were confirmed on both Ultra and culture. 6/10 (60.0%) children with confirmed RR-TB reported no known TB exposure. Of those diagnosed based on exposure, 7/15 (46.7%) were exposed to MDR-TB (two with “trace positive” Ultra but negative cultures), 4/15 (26.7%) to RMR-TB, 2/15 (13.3%) to extensively drug-resistant TB (XDR TB), and 1/15 (6.7%) each to pre-XDR-TB and rifampicin-resistant TB where other drug resistance is unknown. Of the 27 children with drug-resistant TB, five (18.5%) were infants, and seven (25.9%) were CLHIV. All 27 children completed treatment with clinical cure.

### Outcomes

Cohort retention was high with 83% followed up at three months, 78% at the end of treatment, and 70%, at 12 months. The overall mortality was 28/636 (4.4%), and 27/557 (4.9%) amongst children diagnosed with TB. Mortality rates across age groups were as follows; 14/163 (8.6%) for infants, 7/308 (2.3%) for children 1–5 years, and 7/165 (4.2%) for children 5–15 years.

Of 27 deaths amongst children with TB, 13/27 (48.1%) were presumed TB-related based on medical reports. Most deaths (85.2%; 23/27) occurred late, more than four weeks from TB treatment initiation. Adjusted risk ratios (aRR) for mortality; adjusted for stunting, living with HIV, and age less than one year; were highest for children stunted (7.3; 95% CI, 2.2-24.2), followed by CLHIV (3.5; 95% CI, 1.6-7.5), and infants (3.3; 95% CI, 1.6-6.7; Table 3). Similarly, aRR for mortality were high at 4.9 (95% CI, 1.8-13.3; p = 0.002) for WAZ < -2, 2.5 (95% CI, 1.2-5.2; p = 0.020) for CLHIV, and 3.6 (95% CI, 1.7-7.7; p = 0.001) for infants, also adjusted for WAZ < -2, living with HIV, and age less than one year (table not shown).

**Table 3:** Overall mortality rate, and mortality rates, unadjusted risk ratios, and adjusted risk ratios for mortality per risk group, in children treated for PTB.

	Overall, (%)	Mortality Rate, overall and per high-risk group (%)	RR (95% CI)	aRR (95% CI)	p-value
**Overall**		**27/557 (4.9)**			
**Age < 1 year**	137/557 (24.6)	14/137 (10.2)	3.3 (1.6-6.9)	**3.3 (1.6-6.7)**	0.001
CLHIV*	162/555 (29.2)	16/162 (9.9)	3.5 (1.7-7.4)	**3.5 (1.6-7.5)**	0.002
**WAZ** ^**†**^ **<-2**	205/514 (39.9)	19/205 (9.3)	7.2 (2.5-20.8)	**..**	..
**HAZ** ^**‡§**^ **<-2**	244/555 (44.0)	23/244 (9.4)	9.8 (3.0-32.2)	**7.3 (2.2-24.2)**	0.001
**WHZ** ^**‡‖**^ **<-3**	63/405 (15.6)	3/63 (4.8)	1.0 (0.3-3.2)	**..**	..
**BMI-for-age** **<-3** ^**‡**^ **	19/150 (12.7)	5/19 (26.3)	34.5 (4.2-281.4)	**..**	..

Data reported as number (proportion) of the total number for categorical data. Adjusted risk ratios (aRR) for mortality are adjusted for stunting, living with HIV, and age under one year. CLHIV = Children Living with HIV, WAZ = WHO Weight-for-age Z-score, HAZ = WHO Height-for-age, WHZ = WHO Weight-for-length or Weight-for-height Z-score.

* HIV status unknown for two children.

† WAZ calculations based on WHO Z-scores for weight-for-age for children 0–10 years.

‡ Four heights unknown (excluded from HAZ, WHZ, and BMI-for-age calculations).

§ HAZ calculations based on WHO Z-scores for height-for-age for children 0–15 years.

**‖** WHZ calculations based on WHO Z-scores for weight-for-length and weight-for-height for children 0–5 years

**BMI-for-age calculations based on WHO Z-scores for BMI-for-age for children 5–15 years.

## Discussion

This study of Xpert MTB/RIF Ultra on induced sputum in high-risk children showed high diagnostic accuracy and yield for childhood PTB overall, and across high-risk groups; infants, CLHIV, and those with malnutrition. Ultra detected a large proportion of children with negative cultures, many of whom were “trace positive.” Children who were malnourished, followed by CLHIV, and infants, had the highest risk ratio for mortality, with most deaths occurring after the first month of TB treatment.

Sputum induction has been shown to be feasible in young children, and across various settings [5–7,20–22]. Several high burden countries including South Africa have reported the safe use of IS in large cohorts of children [4–8,20–23]. The rate of minor adverse events was very low, and no serious adverse events occurred, which is comparable to other studies [4,22,24].

IS has been shown to provide the highest microbiological diagnostic yield for children compared to other samples, including gastric aspirates, nasopharyngeal aspirates, tongue swabs, and stool [8]. The Eastern Cape, an under-resourced province of South Africa, reports one of the lowest primary healthcare expenditures per capita nationally, with this district providing only 2.5 hospital beds per 10,000 population (under half the national average), and children and adolescents under 19 years making up 30% of the total district population [16,25]. Dora Nginza Hospital, where this study was based, offers both secondary and tertiary care, in addition to caring for children well enough for primary care level where resource limitations preclude primary healthcare services. The participants in this study are likely to be representative of children in low-to-middle income countries as evidenced by their use of public health facilities, and their poor nutritional status and disease burden. Our study demonstrated the successful use of IS in this resource-poor setting, offering a high yield and around 90% sensitivity and specificity for Ultra overall, which was similar across high-risk children.

Over 70% of the first induced sputa were successfully performed by hospital nursing staff following minimal training. This adds to the body of evidence supporting IS as a valuable diagnostic specimen in similar settings, particularly for young children, and in regions with high burdens of TB, HIV, or malnutrition [4–8,20–23]. In addition, this strategy detected a significant number of children with drug-resistant TB disease, many without a known TB exposure, enabling rapid targeted therapy.

Ultra detected Mycobacterium tuberculosis (*Mtb)* in over 90% of children with positive cultures. Ultra further detected *Mtb* in many children with negative cultures (with more than 70% of these, “trace positive”) – increasing overall IS yield from 15.8% to 26.0%; and all of whom, presenting with clinical and radiological signs, were treated for PTB. These data suggest that Ultra, with the ability to detect trace amounts of *Mtb*, may be more sensitive than culture for the microbiological diagnosis of childhood PTB [5,6,20,23]. The increased yield from Ultra may be attributed to a lack of viable bacilli (affected by the host immune response, any anti-TB therapy given, and/or the laboratory decontamination process), which may result in corresponding negative culture results [23]. In adults, Ultra may remain positive following treatment for TB for months thereafter; however, all children were symptomatic and/or had radiological evidence of PTB, all improved on TB treatment, and only a small number had previous TB - with only one child having a TB episode in the prior year [26].

The prevalence of malnutrition was high in this cohort, with CLHIV and infants significantly more at risk for malnutrition, compared to those HIV negative and older, respectively. Children with malnutrition, HIV, or young age, may have impaired immunity, increasing their risk for TB disease and progression [1–3]. As evidence of this, CLHIV and those underweight-for-age had lower TST positivity, while young or malnourished children were at significantly higher risk of being hospitalized.

Children under five years with severe malnutrition (WHZ < -3) had higher yields, and almost twice the risk of having a positive IS1 Ultra compared to those with WHZ ≥ -3 (adjusted for age and HIV status), offering promise for rapid diagnosis in this high-risk group. Malnutrition not only increases risk for TB disease, but may also represent a consequence of disease, potentially implying prolonged disease duration, and more severe pathology [1,27–30]. Adult studies show more severe radiological disease and cavitation in those with severe malnutrition - inferring higher bacillary loads, with animal models suggesting several potential causative mechanisms, including a weakened T-helper 1 cell-mediated immune response, and reduced tumor necrosis factor, interferon-gamma, and nitric oxide synthase expression [27–29]. Micronutrient deficiencies including zinc and vitamin A have also been associated with more severe pulmonary lesions [28–30].

With most deaths occurring after the first month of treatment, and adjusted risk for mortality highest for those malnourished, an urgent need is evident, not only for improved diagnostics in high-risk groups, but for strengthened nutritional rehabilitation, clinical monitoring, psychosocial support, and community engagement [2,3].

Limitations include small sample sizes in sub-groups, limiting statistical power, and a low proportion of children classified as unlikely TB. However, the yield from IS amongst children diagnosed with TB was similarly high compared to other IS-based studies [4,8,19]. Although data were collected at a single center, the sample represented hospitalized children as well as ambulatory cases referred for assessment directly from primary healthcare facilities, offering a more representative sample than those included in hospital-based studies.

Large-scale implementational studies investigating feasibility, acceptability, and diagnostic performance of IS, and impact on overall childhood TB case detection at primary care level, would be important next steps [4,7,8,16–20]. Strengthening sampling at primary care and integrating microbiological data with novel diagnostic tools such as computer-aided radiographic interpretation may improve TB case detection and accuracy for treatment decision algorithms [31]. Improved point-of-care, rapid diagnostics are needed, as a large proportion of children remain as unconfirmed TB cases, as shown in this study.

Childhood TB remains underdiagnosed with high mortality, particularly for infants, malnourished children, and CLHIV. Ultra testing of induced sputum, which provides high yield and diagnostic accuracy, may contribute significantly towards improved diagnosis and outcomes for childhood TB, particularly for high-risk groups.

## Supporting information

S1 Text Supporting Information Methods: Additional methodological detail including details on study setting, definitions, and sampling and microbiological investigations, Supporting Information References. (DOCX)

S1 Table Ultra diagnostic yield and accuracy overall, and across high-risk groups from first IS sample; against culture from any IS sample. (DOCX)

S2 Table Analysis of Ultra “trace positive” results. (DOCX)

S3 Table Comparing children with positive Ultra and negative culture, with those with positive Ultra and positive culture on induced sputum (a per-participant analysis). (DOCX)

S4 Table Ultra yield overall and across risk groups, risk ratios for Ultra positivity across risk groups, and adjusted risk ratios for Ultra positivity per risk category. (DOCX)
