# Supplementary material for: Rapid diagnosis of pulmonary tuberculosis on induced sputum in children in a high burden setting for HIV and malnutrition
Source: PLOS Glob Public Health. 2026 Oct 1;6(10):e0007271. doi: 10.1371/journal.pgph.0007271 (PMC13630226; doi:10.1371/journal.pgph.0007271)
Supplement: Supplementary file 1 [file pgph.0007271.s001.docx]

**Rapid diagnosis of pulmonary TB on induced sputum in children in a high burden setting for HIV and malnutrition**

Juaneta Luiz, Lesley J Workman, Cynthia B Baard, Yekiwe Hlombe, Margaretha Prins, Nomlindo Makubalo, D Jaganath, A Cattamanchi, Mark P Nicol, Heather J Zar

**S1 Text: Supporting Information Methods**

**Setting**

The Eastern Cape, an under-resourced province of South Africa, where this study was based, reports high HIV prevalence rates at 13.7% (all ages), along with high rates of childhood malnutrition (with a severe acute malnutrition case fatality rate of 9.5%) [S1, S2]. Published childhood tuberculosis (TB) data are limited, though high rates of adult microbiological confirmation (865/100,000 in 2015) suggest a hidden childhood epidemic, in a province where children under 10 years make up 19% of the total population [S3, S4].

**Definitions**

For children living with HIV (CLHIV), recent results for CD4 count and percentage were recorded, as well as whether the child was on antiretroviral therapy at time of enrolment.

Under-weight-for-age and severely under-weight-for-age were represented by WAZ<-2 and WAZ<-3 respectively, stunting and severe stunting by HAZ<-2 and HAZ<-3 respectively, and severe malnutrition by WHZ<-3 for children under 5 years, and a BMI-for-age <-3 for children 5-15 years [S5, S6].

Categorization was done using NIH consensus definitions for confirmed (microbiological confirmation with a positive Ultra or culture on a respiratory sample), unconfirmed (clinical diagnosis made without microbiological confirmation), or unlikely TB (negative microbiological investigations, with criteria for unconfirmed tuberculosis not met, and improvement noted on follow-up without TB treatment) [S7]. For baseline characteristics and mortality analyses, microbiological confirmation on an extrapulmonary TB (EPTB) sample was also considered as confirmed TB.

Drug-resistant TB (DR-TB; resistance to any anti-TB drugs) comprised isoniazid mono-resistant TB, and rifampicin-resistant TB (RR-TB): which includes rifampicin-mono-resistant (RMR), multidrug resistant (MDR; resistance to at least rifampicin and isoniazid), pre-extensively drug resistant (pre-XDR; MDR/RR-TB with resistance to fluoroquinolones), and extensively drug resistant (XDR; MDR/RR-TB with resistance to fluoroquinolones and one additional Group A medicine) TB, along with rifampicin-resistance with resistance to other medicines unknown [S5].

**Sampling and microbiological investigations**

For the induced sputum procedure, the equipment used included an appropriately sized pediatric mask and spacer, a bronchodilator (salbutamol) metered dose inhaler sterilized for reuse, a pediatric nebulizer mask kit with tubing, sterile hypertonic saline (5%) with a syringe to draw up the fluid, wall or mobile suction with tubing, a sterile mucus extractor kit (for sputum aspiration) or a sputum specimen container (for expectorated samples), and equipment for pediatric pulse oximetry monitoring. Induced sputa were performed in a dedicated, well-ventilated area. Where possible, children were asked to rinse their mouth and blow their nose with a tissue to clear secretions prior to the procedure. For younger children, the nose was gently wiped clean.

As noted, induction included pre-treatment with the inhaled salbutamol (two metered puffs of 100ug) for bronchodilation. For IS performed by hospital staff where the mask and spacer kit and salbutamol metered dose inhaler were unavailable for daily use, salbutamol was delivered in liquid form diluted with 0.9% normal saline via pediatric nebulizer mask and tubing. This was followed by sterile hypertonic saline delivered with oxygen utilizing the pediatric nebulizer mask and tubing, at a flow rate of 5-8L/min. This allows sputum to be drawn into the larger airways and stimulates a cough to mobilize the secretions into the nasopharynx. For infants (under one year of age), 1-2ml of 5% hypertonic saline was utilized, for children one to two years of age, 2ml was used, and for children over two years of age, 4ml was used. This solution was nebulized until the chamber was empty (usually for two to five minutes), or until the child coughed two to three times in quick succession.

Older children who could adequately expectorate were then instructed on how to cough and expectorate sputum into a sputum specimen container. For younger children who could not adequately expectorate (typically, those under six years of age), the sterile mucus extractor kit was used to suction sputum from the nasopharynx. Firstly, younger children were wrapped in a comfortable position with linen cloth, with the head exposed (covering arms and legs). Following this, the mucus extractor kit was connected to the suction apparatus. The length at which the catheter was to be advanced into the nose was then estimated based on the distance from the nasal ala to the tragus of the ipsilateral ear, and the tubing was pinched at this distance. The suction was initiated at 15-20kPA, and, stabilizing the head, the mucus extractor tubing was advanced gently into the nostril, aiming away from the nasal septum. Once advanced to the marked length, the tubing was released, and gentle suction performed, aiming for collection of at least 1.5ml of sputum.

Pulse oximetry was monitored, pre- and post-procedure, during the nebulization, and if there was any change in the child's respiratory condition. Where possible, two IS were obtained, at least four hours apart. Additional pulmonary and extra-pulmonary samples were taken at the discretion of the treating medical team.

Samples were transported on ice (at 2-8 degrees Celsius) to the local accredited TB laboratory and processed using standardized protocols as per manufacturer instructions. Following decontamination with sodium hydroxide/trisodium citrate and NALC (N-acetyl-l-cysteine), and neutralization with phosphate-buffered saline, centrifuged deposits were re-suspended in 1.5ml of phosphate-buffered saline, and *Xpert MTB/RIF Ultra*, fluorescent acid-fast smear microscopy, and automated liquid culture (*Becton Dickinson Bactec MGIT 960*) were performed. Cultures were incubated for up to 42 days and positive cultures identified by acid-fast staining, followed by either *GenoType MTBDRplus* Line Probe Assay (Hain Lifescience), or, from March 2023, Ultra (if Ultra on the same sample was not already positive). MPT64 antigen testing was performed on all positive cultures for further speciation across the study period. Drug susceptibility testing was performed as required, using molecular methods - initially utilizing *GenoType MTBDRplus* Line Probe Assay, followed by Ultra and *Xpert MTB/RIF XDR* from March 2023. For phenotypic testing, *Becton Dickinson Bactec MGIT 960* was used throughout the study period.

**Supporting Information References**

S1. Ndlovu N, Mokganya M, Blose N, et al. District Health Barometer 2023/24. Durban: Health Systems Trust; March 2025. [Accessed 2025 April 22] Available from: <https://www.hst.org.za/publications/Pages/District-Health-Barometer-202324.aspx>
S2. Zuma K, Zungu NP, Moyo S, et al. The Sixth South African National HIV Prevalence, Incidence and Behaviour Survey, 2022: A Summary Report. HSRC Press: Cape Town; 2025. [Accessed 2025 July 23] Available from: <https://hsrc.ac.za/wp-content/uploads/2024/07/SABSSM_VI_EXEC_REPORT_2PP.pdf>
S3. Nanoo A, Blows L, Ismail F, Koornhof H, Mwansa J, Omar SV, Candy S, Ismail N. Microbiologically Confirmed Pulmonary TB - District. Centre for Tuberculosis and Surveillance Information Management Unit, National Institute for Communicable Disease, 2019. [Accessed 2024 April] Available from: <https://www.nicd.ac.za/wp-content/uploads/2017/05/Article_3.pdf>
S4. Eastern Cape – A Province in South Africa: Wazimap, OpenUp, Media Monitoring Africa; 2022 [accessed 2026 June 6]. Available from: <https://open.wazimap.com/?geo=EC>
S5. World Health Organization. WHO child growth standards: length/height-for-age, weight-for-age, weight-for-length, weight-for-height and body mass index-for-age: methods and development. Geneva: World Health Organisation; 2006 [accessed 2024 October 8].   Available from: <https://www.who.int/publications/i/item/924154693X>
S6. World Health Organization. WHO consolidated guidelines on tuberculosis. Module 5: management of tuberculosis in children and adolescents. Geneva: World Health Organisation 2022.
S7. Graham SM, Cuevas LE, Jean-Phillippe P, Browning R, Casenghi M, Detjen AK, et al. Clinical case definitions for Classification of Intrathoracic Tuberculosis in Children: An Update. *Clin Infect Dis.* 2015;61Suppl 3: S179–187.
