# Supplementary material for: Rapid diagnosis of pulmonary tuberculosis on induced sputum in children in a high burden setting for HIV and malnutrition
Source: PLOS Glob Public Health. 2026 Oct 1;6(10):e0007271. doi: 10.1371/journal.pgph.0007271 (PMC13630226; doi:10.1371/journal.pgph.0007271)
Supplement: Supplementary file 2 [file pgph.0007271.s002.docx]

**S1 Table. Ultra diagnostic yield and accuracy overall, and across high-risk groups from first IS sample; against culture from any IS sample**

| Ultra vs TB Culture per child, on any IS sample | | | | | |
| --- | --- | --- | --- | --- | --- |
| N = 636 |  | **True positive (n)** | **True negative (n)** | **False negative (n)** | **False positive (n)** |
| Ultra overall  (compared to culture) |  | 80 | 491 | 8 | 57 |
| N = 636 | **Yield (%)** | **Sensitivity (%)** | **Specificity (%)** | **PPV (%)** | **NPV (%)** |
| Ultra overall | 137/636 (21.5) | 90.9  (82.9-96.0) | 89.6  (86.7-92.0) | 58.4  (49.7-66.7) | 98.4  (96.9-99.3) |
| By Age per child, on first IS sample | | | | | |
| Age <1 year | 32/166 (19.3) | 72.2  (46.5-90.3) | 86.9  (80.3-91.9) | 40.6  (23.7-59.4) | 96.2  (91.3-98.7) |
| Age ≥1 year and < 5 years | 49/308 (15.9) | 87.1  (70.2-96.4) | 92.1  (88.2-95.0) | 55.1  (40.2-69.3) | 98.5  (96.1-99.6) |
| Age ≥5 years | 44/165 (26.6) | 87.2  (72.6-95.7) | 92.1  (85.9-96.1) | 77.3  (62.2-88.5) | 95.9  (90.6-.98.6) |
| By HIV status per child, on first IS sample | | | | | |
| CLHIV* | 28/168 (16.7) | 87.5  (67.6-97.3) | 95.1  (90.2-98.0) | 75.0  (55.1-89.3) | 97.9  (93.9-99.6) |
| HIV exposed uninfected | 25/155 (16.1) | 87.5  (61.7-98.4) | 92.1  (86.3-96.0) | 56.0  (34.9-75.6) | 98.5  (94.6-99.8) |
| HIV uninfected | 72/311 (23.2) | 81.3  (67.4-91.1) | 87.5  (82.8-91.1) | 54.2  (42.0-66.0) | 96.2  (93.0.2-98.3) |
| By Nutritional Status per child, on first IS sample | | | | | |
| WAZ^†^ <-2 | 46/223 (20.6) | 85.2  (66.3-95.8) | 88.3  (82.9-92.4) | 50.0  (34.9-65.1) | 97.7  (94.3-99.4) |
| WAZ^†^ ≥-2 | 61/367 (16.6) | 81.8  (67.3-91.8) | 92.3  (88.8-94.9) | 59.0  (45.7-71.4) | 97.4  (94.9-98.9) |
| HAZ^‡§^ <-2 | 62/268 (23.1) | 88.1  (74.4-96.0) | 88.9  (84.1-92.7) | 59.7  (46.4-71.9) | 97.6  (94.4-99.2) |
| HAZ^‡§^ ≥-2 | 63/364 (17.3) | 80.4  (66.1-90.6) | 91.8  (88.2-94.6) | 58.7  (45.6-71.0) | 97.0  (94.4-98.6) |
| WHZ^‡‖^ <-3 | 18/68 (26.5) | 100.0  (63.1-100.0) | 83.3  (71.5-91.7) | 44.4  (21.5-69.2) | 100.0  (92.9-100.0) |
| WHZ^‡‖^ ≥-3 | 63/399 (15.8) | 78.0  (62.4-89.4) | 91.3  (87.9-94.0) | 50.8  (37.9-63.6.) | 97.3  (95.0-98.8) |
| BMI-for-age^‡^** <-3 | 8/20 (40.0) | 85.7  (42.1-99.6) | 84.6  (54.6-98.1) | 75.0  (34.9-96.8) | 91.7  (61.5-99.8) |
| BMI-for-age^‡^** ≥-3 | 36/154 (24.84) | 87.5  (71.0-96.5) | 92.9  (86.5-96.9) | 77.8  (60.8-89.9) | 96.3  (90.9-99.0) |
| By Ambulatory vs Hospitalised per child, on first IS sample | | | | | |
| Ambulatory | 49/251 (19.5) | 77.8  (60.8-89.9) | 90.2  (85.5-93.9) | 57.1  (42.2-71.2) | 96.0  (92.3-98.3) |
| Hospitalised | 76/385 (19.7) | 88.5  (76.6-95.6) | 91.0  (87.4-93.8) | 60.5  (48.6-71.6) | 98.1  (95.8-99.3) |
| Multiple risk factors, per child, on first IS sample | | | | | |
| CLHIV*, or WAZ^†^, <-2 or <1yr | 79/380 (20.8) | 83.6  (71.2-92.2) | 89.8  (86.0-92.9) | 58.2  (46.6-69.2) | 97.0  (94.4-98.6) |
| HIV uninfected, and WAZ^†^ >-2, and >1yr | 37/234 (15.8) | 84.0  (63.9-95.5) | 92.3  (87.9-95.6) | 56.8  (39.5-72.9) | 98.0  (94.9-99.4) |
| CLHIV* and WAZ^†^ <-2 | 8/80 (10.0) | 83.3  (35.9-99.6) | 95.9  (88.6-99.2) | 62.5  (24.5-91.5) | 98.6  (92.5-100.0) |
| WAZ^†^ <-2 and <1 yr | 19/82 (23.2) | 75.0  (34.9-96.8) | 82.4  (71.8-90.3) | 31.6  (12.6-56.6) | 96.8  (89.0-99.6) |

Data are reported as frequency and percentages, with 95% Confidence Intervals reported for sensitivity, specificity, positive predictive values, and negative predictive values. IS = induced sputum, PPV = positive predictive value, NPV = negative predictive value, WAZ = weight-for-age Z score, HAZ = height-for-age Z score, WHZ = weight-for-height Z score. CLHIV = children living with HIV.

* HIV status unknown for two children

† WAZ calculations based on WHO Z-scores for weight-for-age for children 0-10 years

‡ Four heights unknown (excluded from HAZ, WHZ, and BMI-for-age calculations)

§ HAZ calculations based on WHO Z-scores for height-for-age for children 0-15 years

**‖** WHZ calculations based on WHO Z-scores for weight-for-length and weight-for-height for children 0-5 years

**BMI-for-age calculations based on WHO Z-scores for BMI-for-age for children 5-15 years
