# Supplementary material for: Rapid diagnosis of pulmonary tuberculosis on induced sputum in children in a high burden setting for HIV and malnutrition
Source: PLOS Glob Public Health. 2026 Oct 1;6(10):e0007271. doi: 10.1371/journal.pgph.0007271 (PMC13630226; doi:10.1371/journal.pgph.0007271)
Supplement: Supplementary file 3 [file pgph.0007271.s003.docx]

**S2 Table. Analysis of Ultra “trace positive” results**

| On Any Induced Sputum* | | | | |
| --- | --- | --- | --- | --- |
|  | **Ultra positive; non-“trace positive”**  **(% of column total)**  **[74/636; 11.6%]** | **Ultra “trace positive”**  **(% of column total)**  **[63/636; 9.9%]** | **Ultra negative**  **(% of column total)**  **[499/636; 78.5%]** | **Totals** |
| Culture positive | 58 (78.4) | 22 (34.9) | 8 (1.6) | 88 |
| Culture negative | 16 (21.6) | 41 (65.1) | 491 (98.4) | 548 |
| Total | **74** | **63** | **499** | 636 |
| Features of children with a positive Ultra (non-“trace positive”) vs Ultra “trace positive” on Any IS* | | | | |
|  | **Ultra positive; non-“trace positive” (n=74)** | **Ultra “trace positive”**  **(n=63)** | **p-value** |  |
| Median age (IQR) months | 40.3  (12.2-95.3) | 29.4  (10.6-62.2) | 0.325 |  |
| Age <1 year | 18 (24.3%) | 17 (27.0%) | 0.722 |  |
| CLHIV^†^ | 18 (24.3%) | 14 (22.2%) | 0.772 |  |
| WAZ<-2^‡^ (n=117) | 29 (46.8%) | 21 (38.2%) | 0.348 |  |
| HAZ<-2^§‖^ | 40 (54.1%) | 26 (41.3%) | 0.136 |  |
| WHZ<-3^§**^ (n=88) | 8 (18.6%) | 11 (24.4%) | 0.506 |  |
| BMI-for-age  <-3^§††^ (n=49) | 8 (25.8%) | 1 (5.6%) | 0.078 |  |

Data reported as number (proportion) of the total number for categorical data, and median (IQR) for continuous data. IS = Induced sputum, IQR = interquartile range, CLHIV = Children Living with HIV, WAZ = WHO weight-for-age Z-score, HAZ = WHO height-for-age Z-Score, WHZ = WHO weight-for-length or weight-for-height Z-score.

*Any Induced Sputum – taking the most positive result from all IS performed for each child

† HIV status unknown for two children

‡ WAZ calculations based on WHO Z-scores for weight-for-age for children 0-10 years

§ Four heights unknown (excluded from HAZ, WHZ, and BMI-for-age calculations)

**‖** HAZ calculations based on WHO Z-scores for height-for-age for children 0-15 years

**WHZ calculations based on WHO Z-scores for weight-for-length and weight-for-height for children 0-5 years

††BMI-for-age calculations based on WHO Z-scores for BMI-for-age for children 5-15 years
