# Supplementary material for: Rapid diagnosis of pulmonary tuberculosis on induced sputum in children in a high burden setting for HIV and malnutrition
Source: PLOS Glob Public Health. 2026 Oct 1;6(10):e0007271. doi: 10.1371/journal.pgph.0007271 (PMC13630226; doi:10.1371/journal.pgph.0007271)
Supplement: Supplementary file 4 [file pgph.0007271.s004.docx]

**S3** **Table.** **Comparing children with positive Ultra and negative culture, with those with positive Ultra and positive culture on induced sputum (a per-participant analysis)**

|  | Ultra positive and Culture negative  57/137 (41.6%) | Ultra positive and Culture positive  80/137 (58.4%) | p-value |
| --- | --- | --- | --- |
| Median Age (IQR) months | 23.1 (8.2-47.5) | 49.0 (18.8-114.9) | 0.001 |
| CLHIV* (%) | 9/57 (15.8%) | 23/80 (28.8%) | 0.077 |
| WAZ^†^ <-2 (%) | 25/54 (46.3%) | 25/63 (39.7%) | 0.471 |
| HAZ^‡§^ <-2 (%) | 27/57 (47.4%) | 39/80 (48.8%) | 0.873 |
| WHZ^‡‖^ <-3 (%) | 11/45 (24.4%) | 8/43 (18.6%) | 0.506 |
| BMI-for-age^‡^** <-3 (%) | 7/37 (18.9%) | 2/12 (16.7) | 0.861 |

Data reported as number (proportion) of the total number for categorical data, and median (IQR) for continuous data. IQR = interquartile range, CLHIV = Children Living with HIV, WAZ = WHO Weight-for-age Z-score, HAZ = WHO Height-for-age Z-score, WHZ = WHO Weight-for-length or weight-for-height Z-score; reported as number (%), p-values were generated using Wilcoxon rank-sum test and Chi-squared test of equal proportions.

* HIV status unknown for two children

† WAZ calculations based on WHO Z-scores for weight-for-age for children 0-10 years

‡ Four heights unknown (excluded from HAZ, WHZ and BMI-for-age calculations)

§ HAZ calculations based on WHO Z-scores for height-for-age for children 0-15 years

**‖** WHZ calculations based on WHO Z-scores for weight-for-length and weight-for-height for children 0-5 years

**BMI-for-age calculations based on WHO Z-scores for BMI-for-age for children 5-15 years
