# Supplementary material for: Rapid diagnosis of pulmonary tuberculosis on induced sputum in children in a high burden setting for HIV and malnutrition
Source: PLOS Glob Public Health. 2026 Oct 1;6(10):e0007271. doi: 10.1371/journal.pgph.0007271 (PMC13630226; doi:10.1371/journal.pgph.0007271)
Supplement: Supplementary file 5 [file pgph.0007271.s005.docx]

**S4 Table: Ultra yield overall and across risk groups, risk ratios (RR) for Ultra positivity across risk groups, and adjusted risk ratios (aRR) for Ultra positivity per risk category**

|  | **Ultra positive on IS1** | **RR**  **(95% CI)** | **p-value** | **aRR**  **(95% CI)** | **p-value** |
| --- | --- | --- | --- | --- | --- |
| **Overall** | **125/636 (19.7)** | .. | .. | .. | .. |
| **Age < 1 year** | 32/163 (19.6) | .. | .. | Ref. | .. |
| **Age ≥1 year and < 5 years** | 49/308 (15.9) | 0.8 (0.5-1.2) | 0.307 | **0.8 (0.6-1.2)** | **0.386** |
| **Age ≥5 years** | 44/165 (26.7) | 1.4 (0.9-2.0) | 0.134 | .. | .. |
| **CLHIV*** | 28/168 (16.7) | 0.8 (0.5-1.2) | 0.25 | **0.3 (0.1-0.7)** | **0.003** |
| **HIV uninfected** | 97/466 (20.8) | .. | .. | Ref. | .. |
| **WAZ**^†^ **<-2** | 46/223 (20.6) | 1.2 (0.9-1.8) | 0.220 | .. | .. |
| **WAZ**^†^ **≥2** | 61/367 (16.6) | .. | .. | .. | .. |
| **HAZ**^‡§^ **<-2** | 62/268 (23.1) | 1.2 (0.9-1.8) | 0.238 | .. | .. |
| **HAZ**^‡§^ **≥-2** | 63/364 (17.3) | .. | .. | .. | .. |
| **WHZ**^‡‖^ **<-3** | 18/68 (26.5) | 1.7 (1.1-2.6) | 0.027 | **1.7 (1.1-2.6)** | **0.027** |
| **WHZ**^‡‖^ **≥-3** | 63/399 (15.8) | .. | .. | Ref. | .. |
| **BMI-for-age**^‡^** **<-3** | 8/20 (40.0) | 1.6 (0.9-3.0) | 0.125 | .. | .. |
| **BMI-for-age**^‡^** **≥-3** | 36/145 (24.8) | .. | .. | .. | .. |

Adjusted risk ratios (aRR) for Ultra positivity are adjusted for the effects of WHZ, HIV status, and age, for children 0-5 years old (as WHO weight-for-height Z scores are limited to children 0-5 years old). CLHIV = Children Living with HIV, WAZ = WHO weight-for-age Z-score, HAZ = WHO height-for-age Z-Score, WHZ = WHO weight-for-length or weight-for-height Z-score.

* HIV status unknown for two children

† WAZ calculations based on WHO Z-scores for weight-for-age for children 0-10 years

‡ Four heights unknown (excluded from HAZ, WHZ and BMI-for-age calculations)

§ HAZ calculations based on WHO Z-scores for height-for-age for children 0-15 years

**‖** WHZ calculations based on WHO Z-scores for weight-for-length and weight-for-height for children 0-5 years

** BMI-for-age calculations based on WHO Z-scores for BMI-for-age for children 5-15 years
